# Progressive multifocal leukoencephalopathy in a patient with novel mutation in the *RAC2* gene: a case report

**DOI:** 10.1186/s13256-022-03333-7

**Published:** 2022-06-11

**Authors:** Sima Bahrami, Saba Arshi, Mohammad Nabavi, Mohammad Hassan Bemanian, Morteza Fallahpour, Afshin Rezaeifar, Sima Shokri

**Affiliations:** Department of Allergy and Clinical Immunology, Iran University of Medical Sciences, 1445613131 Tehran, Iran

**Keywords:** Progressive multifocal leukoencephalopathy, Common variable immunodeficiency, RAC2 mutation

## Abstract

**Background:**

Progressive multifocal leukoencephalopathy is a rare demyelinating disease that is often secondary to lytic destruction of oligodendrocytes and, to a lesser extent, to astrocytes’ response to human neurotrophic John Cunningham polyomavirus. Any underlying congenital disorder of primary or secondary immunodeficiency may predispose to virus infection and possible invasion of the brain. We present the first reported case of progressive multifocal leukoencephalopathy due to a mutation in the *RAC2* gene.

**Case presentation:**

We describe the case of a 34-year-old Iranian man with recurrent infections from the age of 2 years, along with other disorders such as nephritic syndrome, factor XI deficiency, and hypogammaglobulinemia. He was treated regularly with intravenous immunoglobulin from the age of 10 years with a diagnosis of common variable immune deficiency. Genetic testing confirmed a novel homozygous mutation in the *RAC2* gene in the patient. Owing to the onset of neurological symptoms a few months ago, the patient was completely avaluated, which confirmed the diagnosis of PML. Despite all efforts, the patient died shortly after progression of neurological symptoms.

**Conclusions:**

According to previous studies, progressive multifocal leukoencephalopathy has been associated with 26 cases of primary immunodeficiency. Our patient presents a new case of primary immunodeficiency with progressive multifocal leukoencephalopathy. Accurate examination of these cases can help us to gain insight into the immune response to John Cunningham virus and better treat this potentially deadly disease.

## Introduction

Progressive multifocal leukoencephalopathy (PML) is a severe and rare demyelinating disorder. It affects white matter and, to a lesser extent, gray matter, including the basal ganglia and thalamus. The disease is caused by a reaction to human neurotrophic John Cunningham virus (JCV), which is one of the most common viral infections in communities [1]. People with normal immune systems are able to successfully control this infection. Oligodendrocytes are the main target of JCV in the brain and, to a lesser extent, astrocytes. Focal or extensive myelin destruction occurs following the lytic degradation of these cells and the release of viral fragments [2].

Adaptive immunity, especially cellular immunity, plays a significant role in controlling this infection, and impairment of the immune system, either primary or secondary, predisposes to reactivation of the virus [3]. PML was first described in 1958 in three patients with hematologic malignancy [4] but was broadly defined in 1980 during the acquired immunodeficiency syndrome (AIDS) epidemic. During the previous decades, the treatment of cancer or rheumatologic disorders by using monoclonal antibodies once again increased the prevalence of secondary PML [3].

PML is seen as a rare complication of several types of primary immunodeficiency (PID), most of which are in the form of combined immunodeficiency. Twenty-six cases of PID with PML have been reported, of which some, such as STAT1 gain of function (GOF), Wiskott–Aldrich syndrome, and DOCK8 deficiency, have been reported in more than one patient, but disorders such as X-linked agammaglobulinemia, CD40 ligand (CD40L) deficiency, purine nucleoside phosphorylase (PNP) deficiency, adenosine deaminase (ADA) deficiency, and immunodeficiency-centromeric instability-facial anomalies syndrome (ICF syndrome), were found in only a single case [5]. In seven patients, PML has been reported as a complication of combined immunodeficiency. Disorders such as hypogammaglobulinemia, hyper-Immunoglobulin (ig)E syndrome, and, in one case, hyper-IgM syndrome have been associated with PML [6]. Unfortunately, PML prognosis is very disappointing; therefore, early diagnosis of disease in the presence of predisposing conditions such as primary or secondary immunodeficiency and early initiation of treatment may be effective in increasing patients’ survival. In this report, we are the first to describe PML in a patient with an early diagnosis of common variable immune deficiency (CVID) and known mutations in the *RAC2* gene.

## Case presentation

Our patient is a 34-year-old Iranian man of Gilaki ethnicity from consanguineous parents, who was 2 years old at the onset of the disease, with recurrent sinopulmonary infections, poor weight gain, urticaria, and sinusitis at age of 7 years. Owing to the low serum level of IgA, he was first diagnosed with selective IgA deficiency. At the age of 8 years, he developed post-infection glomerulonephritis and then was treated as a case with nephritic syndrome. Lack of factor XI is another complication that was diagnosed at the age of 10 years. At the same age, owing to a decrease of IgM–IgG along with recurrent infections, he was diagnosed with CVID, and since then he has undergone regular intravenous immunoglobulin (IVIG) therapy. Also, he was treated separately for other comorbidities, including bronchiectasis, hypothyroidism, and growth hormone deficiency.

As his sister presented with similar immunodeficiency manifestations, they were genetically examined by whole-exome sequencing (WES) analysis. A novel homozygous nonsense mutation in codon 56 (W56X) was detected on the *RAC2* gene, which was confirmed by Sanger sequencing [7].

**Fig. 1:**
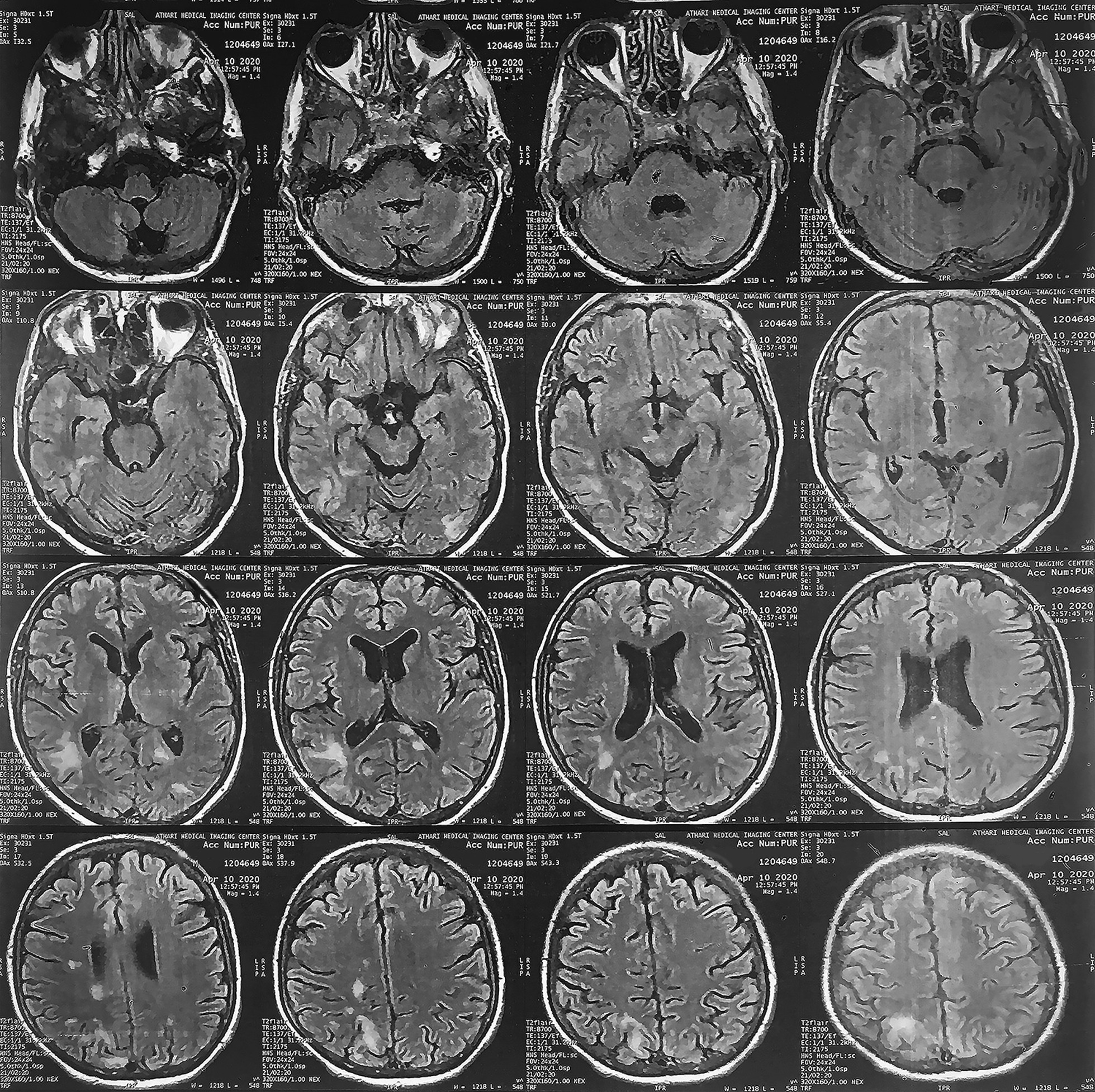
Magnetic resonance imaging of the brain, showing subcortical signal changes in the left occipital and right temporoparietal area

**Fig. 2:**
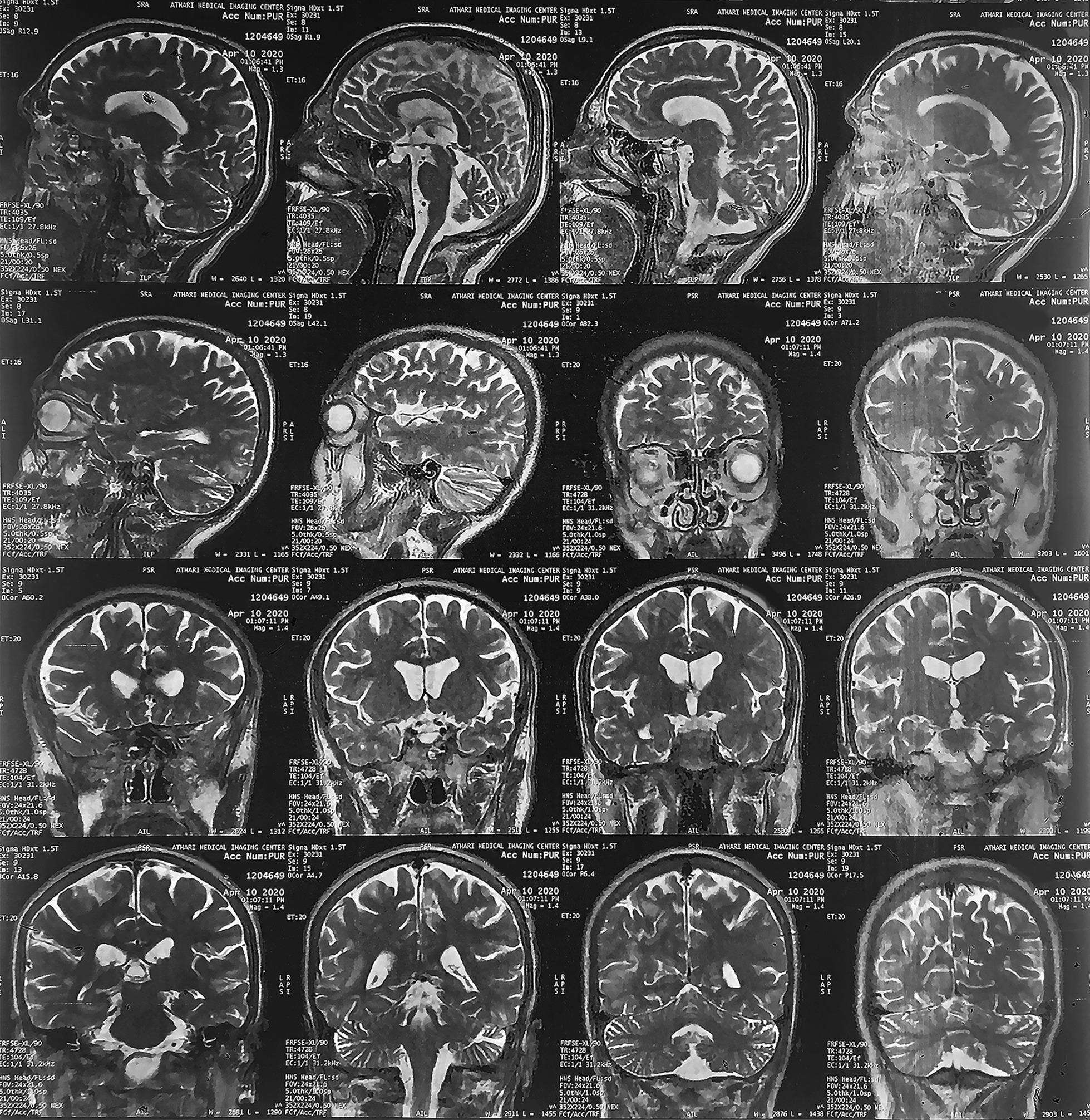
Magnetic resonance imaging of the brain, indicting subcortical signal changes in the left side of the splenium corpus callosum and the right side of the thalamus

About 4 months ago, the patient was first diagnosed with nausea and headache, which gradually worsened along with progressive blurred vision, paresis, and paresthesia on the left side of the body. On brain magnetic resonance imaging (MRI), subcortical lesions in the left occipital and right temporoparietal area (Fig. 1), as well as the involvement of parts of the corpus callosum and thalamus (Fig. 2), suggested the possibility of diseases involving cerebral white matter, such as posterior reversible encephalopathy syndrome (PRES) or PML. To confirm the diagnosis, the patient underwent a more accurate workup. The combination of clinical and radiological findings, along with positive polymerase chain reaction (PCR) for JCV in a cerebrospinal fluid (CSF) sample, confirmed the PML diagnosis. Other tests that were performed on the CSF sample, including glucose, protein, and inflammatory cell counts, were reported to be normal. To determine the risk factors for PML, other than PID, in the patient, human immunodeficiency virus (HIV) Ribonucleic acid (RNA) Polymerase chain reaction (PCR) testing was performed, which was reported negative. In general, the patient’s PID can be considered the most important risk factor for PML. Despite the treatments, our patient died within a month of diagnosis. He was the first case reported with PML due to mutations in the *RAC2* gene.

## Discussion

Human neurotrophic polyomavirus JCV is considered the most important cause of diffuse demyelinating disease, such as PML [6]. This virus is first transmitted through the nose and mouth and then transmitted to the bone marrow and kidneys through infected lymphocytes [8]. Under these conditions, the virus can remain in the latent phase for many years. It is clear that, under the conditions of dysfunction of the immune system, the invading virus increasingly causes uncontrolled infection throughout the body.

Although the exact mechanism of the immune response to JCV is still unclear, a review of the 26 cases of PID associated with PML helps solve this complex puzzle. For example, a report of four cases with PML who had gain-of-function (GOF) mutations in STAT-1 revealed the major role of STAT-1 in controlling interferon (IFN) responses. Accordingly, it can be concluded that IFNs, especially gamma interferon (IFN-γ), play a significant role in controlling JCV [9]. As another hypothesis in patients with GOF mutations in STAT-1, increased expression of programmed cell death protein ligand 1 (PD-L1) on T cells leads to anergy and dysfunction of these cells, which in turn predisposes to PML [6]. The association of disorders such as DOCK8 deficiency [10] and hyper-IgE syndrome [11] with PML suggests a possible role for IL-17 in controlling the virus in normal individuals. Our report regarding the first PML case with *RAC2* deficiency mutation provoked us to investigate the possible roles of this gene in the response of the immune system to JCV. RAC2 protein, as a member of the Rho GTPase family, plays a key role in the regulation of the cellular process of neutrophils in the activity of cytoskeleton actin dynamics, chemotaxis of neutrophils, cell migration, and NADPH oxidase activity [12].

Although our patient and his sibling had no symptoms in favor of neutrophil dysfunction despite having mutations in the same gene during infancy, the first manifestations at older ages can be attributed to their hypogammaglobulinemia, which suggests a significant role of the *RAC2* gene in the development and activation of B and T cells. In the sample that was taken from the patient, a decrease in chemotaxis, as well as in the number and morphology of granules in neutrophils, confirmed serious dysfunction in these cells, but the patient did not have a specific manifestation in favor of neutrophil disorder. Nevertheless, immunophenotyping of lymphocytes in the patient indicated B-cell lymphopenia and abnormalities in T-cell subpopulations, with reversed ratio of CD4/CD8 T cells, decreased percentages of naive CD4, and CD8 T cells, and regulatory T and recent thymic emigrant cells [12]. Therefore, it may be concluded that the patient had a combined disorder of B cells and T cells, which predisposed him to reactivation of latent JCV infection and PML. Cellular immunity plays a very important role in controlling JCV infection. The role of humoral immunity is not exactly known; in the patient of the current study, PCR for JCV was performed on a sample taken from CSF to confirm the diagnosis of PML [13].

Regarding PML treatment, it is important to pay attention to a few general strategies after making a correct and timely diagnosis. The first point is to prevent the virus from entering the cells, and the next point is to improve the immune response against the virus and treat predisposing underlying diseases. In the reported patient, with the aim of improving the immune response, firstly, the dose of IVIG was increased. The measured IgG level of the patient was reported to be 1058 mg/dL during hospitalization. Secondly, mirtazapine was used as an antiviral drug to block one of the virus receptors (5HTR2) in the host cells. Pembrolizumab as a PD-L1 inhibitor was another option in the treatment of the patient, although, unfortunately, owing to the high cost, despite the great efforts of the family and medical staff, it was not possible to prepare and prescribe this drug for the patient. Our patient did not respond to any of the treatments and suffered from the progression of neurological symptoms, and eventually died.

## Conclusion

PML has been reported as a fatal complication in 26 patients with PID due to various gene mutations. Our patient is the first case reported with PML due to mutations in the *RAC2* gene. Early diagnosis and treatment are very important in the prognosis of PML. Owing to the rarity of the complication, it seems necessary to report all cases of it.

## Data Availability

The data used to support the findings of this study are available from the corresponding author upon request.

## Abbreviations

PML: Progressive multifocal leukoencephalopathy
JCV: JC virus
PID: Primary immunodeficiency
CVID: Common variable immune deficiency
GOF: Gain of function
PNP: Purine nucleoside phosphorylase
ADA: Adenosine deaminase
WES: Whole-exome sequencing
PRES: Posterior reversible encephalopathy syndrome
IVIG: Intravenous immunoglobulin
5HTR2: 5-Hydroxytryptamine receptor 2

## References

[1] Bellizzi A, Anzivino E, Rodio DM, Palamara AT, Nencioni L, Pietropaolo V. New insights on human polyomavirus JC and pathogenesis of progressive multifocal leukoencephalopathy. Clin Dev Immunol. 2013;2013:1–17. 10.1155/2013/839719. PMC3652120 23690827

[2] Cavanagh JB, Greenbaum D, Marshall AHE, Rubinstein LJ. Cerebral demyelination associated with disorders of the reticuloendothelial system. Lancet. 1959;274(7102):524–9. 10.1016/s0140-6736(59)91774-x 13808583

[3] Bartt RE, Aksamit A. Neuro-infectious diseases, an issue of neurologic clinics e-book. Neurol Clin. 2018. 10.1016/S0733-8619(18)31262-3.

[4] Astrom KE, Mancall EL, Richardson Jr. EP, Zu Rhein G. Progressive multifocal leukoencephalopathy (PML): a 50-years old disease. Brain. 1958;81:93–111. 10.1093/brain/81.1.93 13523006

[5] Hadjadj J, Guffroy A, Delavaud C, Taieb G, Meyts I, Fresard A, Streichenberger N, *et al*. Progressive multifocal leukoencephalopathy in primary immunodeficiencies. J Clin Immunol. 2019;39(1):55–64. 10.1007/s10875-018-0578-8 30552536

[6] Zerbe CS, Marciano BE, Katial RK, Santos CB, Adamo N, Hsu AP, Hanks ME, *et al*. Progressive multifocal leukoencephalopathy in primary immune deficiencies: Stat1 gain of function and review of the literature. Clin Infect Dis. 2016;62(8):986–94. 10.1093/cid/civ1220 PMC4803104 26743090

[7] Alkhairy OK, Rezaei N, Graham RR, Abolhassani H, Borte S, Hultenby K, Chenglin W, Aghamohammadi A, Williams DA, Behrens TW, Hammarström L, Pan-Hammarström Q. RAC2 loss-of-function mutation in 2 siblings with characteristics of common variable immunodeficiency. J Allergy Clin Immunol. 2015;135(5):1380-1384.e5. 10.1016/j.jaci.2014.10.039. PMC4426222 25512081

[8] Dörries K, Vogel E, Günther S, Czub S. Infection of human polyomaviruses JC and BK in peripheral blood leukocytes from immunocompetent individuals. Virology. 1994;198(1):59–70. 10.1006/viro.1994.1008 8259683

[9] De-Simone FI, Sariyer R, Otalora Y-L, Yarandi S, Craigie M, Gordon J, Sariyer IK. IFN-gamma inhibits JC virus replication in glial cells by suppressing T-antigen expression. PLoS ONE. 2015;10(6):e0129694. 10.1371/journal.pone.0129694. PMC4465661 26061652

[10] Day-Williams AG, Sun C, Jelcic I, McLaughlin H, Harris T, Martin R, Carulli JP. Whole genome sequencing reveals a chromosome 9p deletion causing DOCK8 deficiency in an adult diagnosed with hyper IgE syndrome who developed progressive multifocal leukoencephalopathy. J Clin Immunol. 2015;35(1):92–6. 10.1007/s10875-014-0114-4 PMC4306731 25388448

[11] Angelini L, Pietrogrande MC, Delle Piane MR, Zibordi F, Cinque P, Maccagnano C, Vago L. Progressive multifocal leukoencephalopathy in a child with hyperimmunoglobulin E recurrent infection syndrome and review of the literature. Neuropediatrics. 2001;32(5):250–5. 10.1055/s-2001-19119 11748496

[12] Troeger A, Williams DA. Hematopoietic-specific Rho GTPases Rac2 and RhoH and human blood disorders. Exp Cell Res. 2013;319(15):2375–83. 10.1016/j.yexcr.2013.07.002 PMC3997055 23850828

[13] Berger JR, Aksamit AJ, Clifford DB, Davis L, Koralnik IJ, Sejvar JJ, Bartt R, Major EO, Nath A. PML diagnostic criteria: consensus statement from the AAN neuroinfectious disease section. Neurology. 2013;80(15):1430–8. 10.1212/WNL.0b013e31828c2fa1 PMC3662270 23568998

